# Bilateral Globus Pallidus Lesions and Delayed Hypoxic Encephalopathy Induced by Overuse of Transdermal Fentanyl Patches

**DOI:** 10.7759/cureus.71484

**Published:** 2024-10-14

**Authors:** Hiroaki Maeda, Koji Hayashi, Tomoki Ogawa, Asuka Suzuki, Yuka Nakaya, Toyoaki Miura, Mamiko Sato, Yasutaka Kobayashi

**Affiliations:** 1 Rehabilitation Medicine, Fukui General Hospital, Fukui, JPN; 2 Physical Therapy Rehabilitation, Fukui General Hospital, Fukui, JPN; 3 Health Science, Fukui Health Science University, Fukui, JPN

**Keywords:** bilateral globus pallidus lesions, fentanyl, globus pallidus lesions, hypoxic encephalopathy, neurological side effects, side‐effect, transdermal patch of fentanyl

## Abstract

We describe a rare case of bilateral globus pallidus lesions (BPL) and delayed hypoxic encephalopathy (DHE) induced by the overuse of transdermal fentanyl patches. The patient was a 54-year-old woman, who had a history of unexplained, intractable anal pain, for which several medications were prescribed, but with very limited effectiveness. Four days prior to admission, she was newly prescribed transdermal fentanyl patches at a dose of 4 mg/day. She developed impaired consciousness and respiratory distress after applying more than 10 fentanyl patches across her body. Brain computed tomography (CT) revealed a lesion in the left globus pallidus. She was treated with naloxone and mechanical ventilation in the intensive care unit and regained consciousness, being discharged from the hospital on day 9. However, she later experienced cognitive and behavioral changes, prompting a return to her previous hospital. Brain magnetic resonance imaging (MRI) revealed BPL with hyperintensities on T2-weighted imaging. After readmission, she again developed impaired consciousness and became fully dependent on care. Although her consciousness gradually improved, she developed higher brain dysfunction, myoclonus, and parkinsonism. A follow-up brain MRI two months after the initial onset showed abnormal signals in the deep white matter bilaterally, along with BPL, with hyperintensities in limited areas on T1-weighted imaging and widespread hyperintensities on T2-weighted imaging. The diagnosis of DHE was based on the extent of bilateral white matter lesions. With rehabilitation treatment, her condition improved to the point where she could manage daily life, though attention and memory impairments persisted.

Transdermal fentanyl patches are widely used in clinical practice due to their high efficacy and safety. However, fentanyl overuse has been associated with BPL and DHE, although the exact mechanism remains unclear. This report highlights that even with transdermal administration, overdose can lead to severe neurological side effects.

## Introduction

Fentanyl, a synthetic μ-opioid receptor agonist, is approved for treating moderate to severe pain [1]. It has a faster onset of action and is approximately 100 times more potent than morphine [1]. Due to its high potency compared to other opioids, fentanyl and its structurally related synthetic counterparts have raised significant concerns in both the public and medical communities, primarily due to the disproportionately high rates of overdose fatalities [2,3]. One form of fentanyl, the transdermal patch, is frequently used in palliative care and for managing chronic pain [4]. The transdermal route bypasses the liver’s first-pass metabolism, increasing fentanyl's bioavailability to 90%, which allows for lower doses to be used and reduces the likelihood of adverse effects [3]. Transdermal fentanyl patches are commonly used in routine clinical practice due to their simplicity and ease of use, and it has the advantage of having few side effects (except constipation) due to minimal changes in blood levels [4]. In this report, we describe a case of bilateral globus pallidus lesions (BPL) and delayed hypoxic encephalopathy (DHE) resulting from an overuse of transdermal fentanyl patches.

## Case presentation

**Figure 1:**
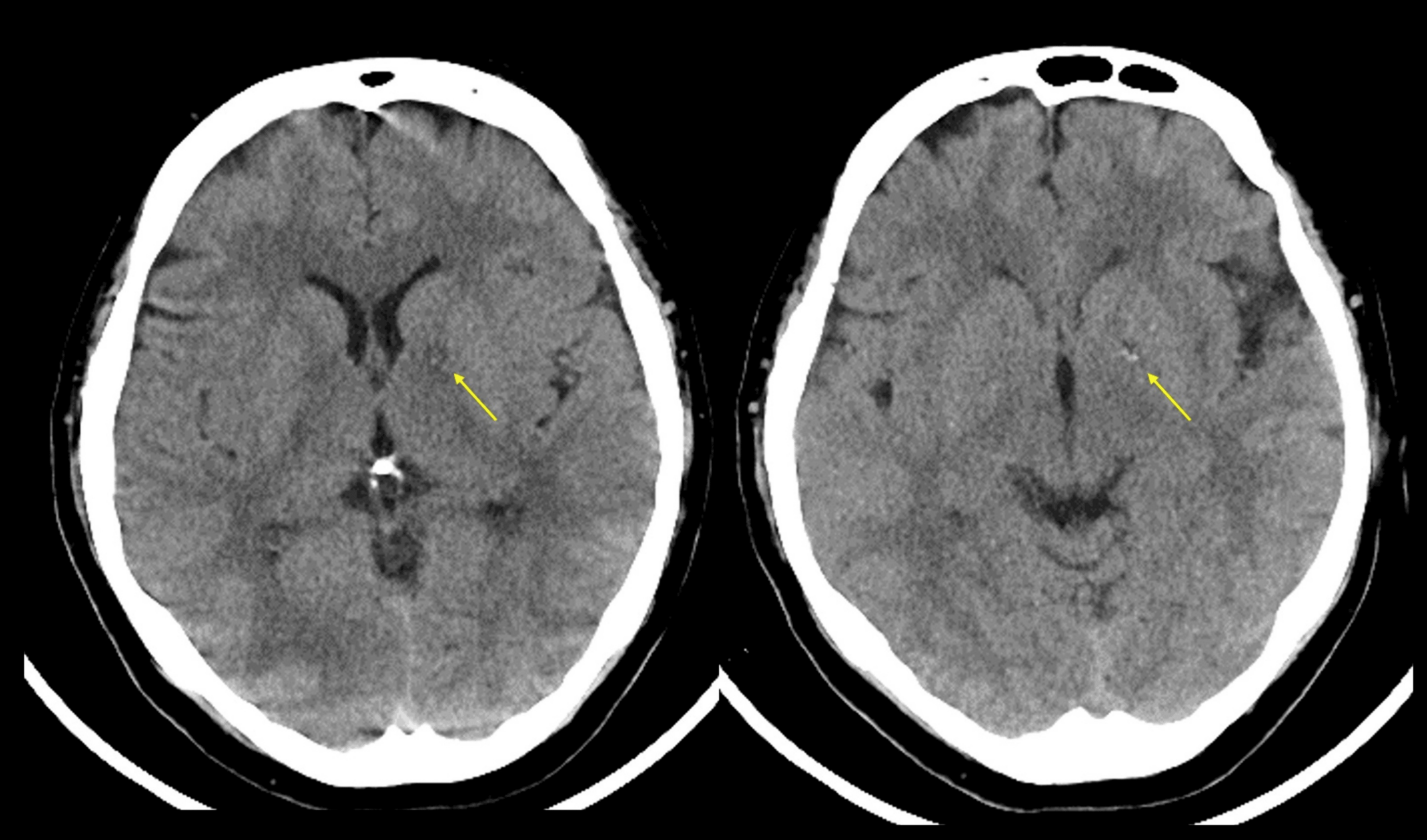
The result of brain CT. Brain CT on initial onset showing a low-density area mixed with a high-density area in the left globus pallidus. CT, computed tomography

**Figure 2:**
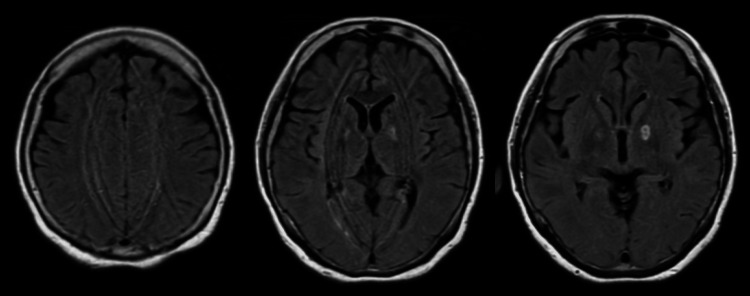
The result of T2-FLAIR brain MRI one month after initial onset. T2-FLAIR brain MRI showing BPL with hyperintensities on T2-FLAIR weighted imaging and no white matter lesion. FLAIR, fluid-attenuated inversion recovery; MRI, magnetic resonance imaging; BPL, bilateral globus pallidus lesions

A 54-year-old woman had a history of unexplained, intractable anal pain, for which several medications were prescribed, but with very limited effectiveness. Four days prior to admission, she was newly prescribed transdermal fentanyl patches at a dose of 4 mg/day. She developed impaired consciousness and respiratory distress after applying more than 10 fentanyl patches across her body. She was transported to another hospital, where several tests and treatments were performed at the previous facility. Brain computed tomography (CT) showed a low-density area mixed with a high-density area in the left globus pallidus (Figure 1), and hypoxic encephalopathy was diagnosed. She received naloxone and rigorous respiratory management with the use of a ventilator. She regained consciousness and was discharged on day 9. After discharge, the transdermal fentanyl patch was discontinued. Subsequently, she experienced unusual episodes, such as causing a self-inflicted car accident with no memory of it, and exhibiting abnormal behavior like wearing a mask as a sock, leading to her hospitalization one month after the initial onset. Brain magnetic resonance imaging (MRI) on the next day revealed BPL with hyperintensities on T2-fluid attenuated inversion recovery (FLAIR) weighted imaging and no white matter lesion (Figure 2).

**Figure 3:**
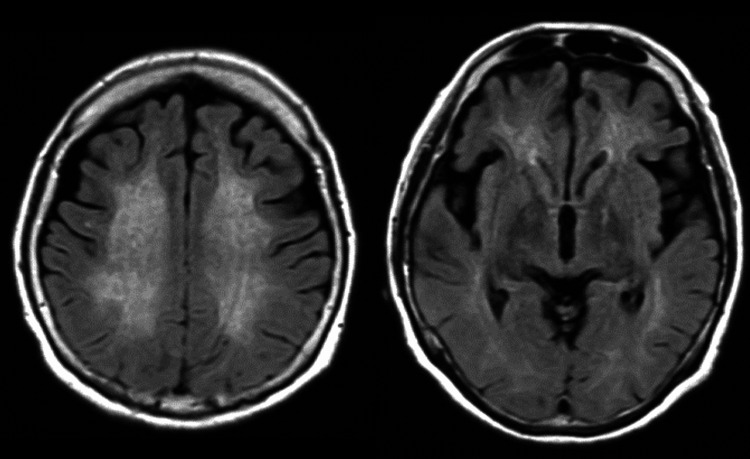
The result of T2-FLAIR brain MRI two months after onset. T2-FLAIR brain MRI showing bilateral, symmetrical hyperintensities in the deep white matter. These lesions were not noted by MRI one month after onset. FLAIR, fluid-attenuated inversion recovery; MRI, magnetic resonance imaging

**Figure 4:**
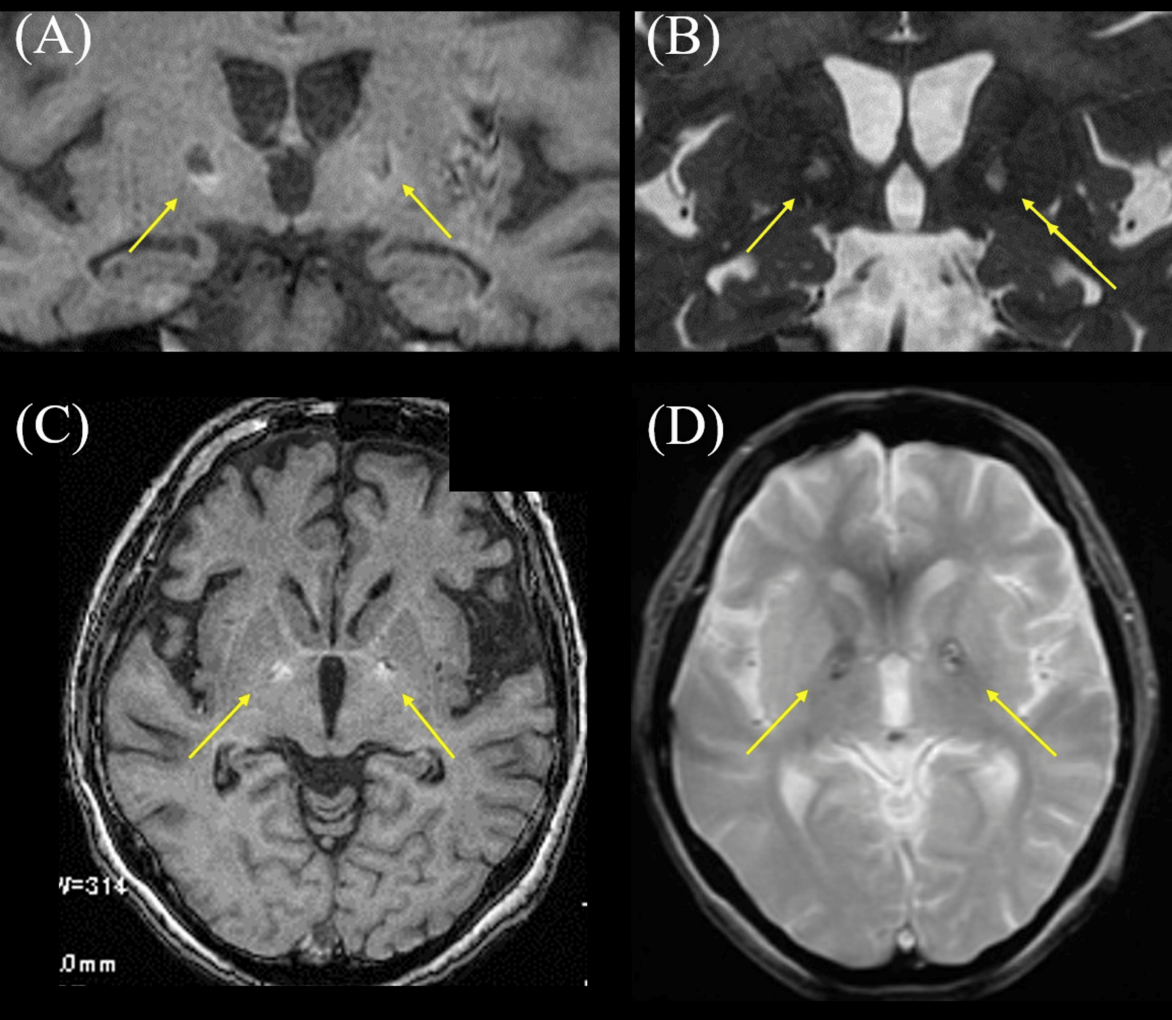
The result of brain MRI 10 months after onset. (A,C) T1-weighted brain MRI showing BPL with mixed hyperintensities and hypointensities on T1-weighted imaging. (B) T2-weighted brain MRI showing BPL with hyperintensities. (D) T2*-weighted brain MRI showing BPL with patchy hypointensities. MRI, magnetic resonance imaging; BPL, bilateral globus pallidus lesions

After admission, she again developed impaired consciousness and became fully dependent on care. Although her level of consciousness gradually improved, she developed higher brain dysfunction, myoclonus, and parkinsonism. A follow-up MRI two months after initial onset showed abnormal signals in the deep white matter on both sides, in addition to BPL, with hyperintensity in limited areas on T1-weighted imaging and hyperintensity in almost all areas on T2-weighted imaging (Figure 3). We diagnosed the patient with DHE based on the extent of bilateral white matter lesions. As a result of the rehabilitation treatment, although attention and memory impairments remained, her condition improved to a level where she could manage daily life, and she was discharged home eight months after onset. A follow-up brain MRI 10 months after initial onset revealed BPL with mixed hyperintensities and hypointensities on T1-weighted imaging, hyperintensities on T2-weighted imaging, and patchy hypointensities on T2*-weighted imaging (Figure 4).

## Discussion

We describe a case with unique brain imaging findings following the overuse of transdermal fentanyl patches. She compulsively overused transdermal fentanyl patches, which had been newly prescribed at a dose of 4 mg/day, four days prior to admission. The patient developed abnormal behavior, disturbed consciousness, myoclonus, and parkinsonism. Brain CT and MRI initially revealed BPL, followed by the appearance of hypoxic encephalopathy two months later. The patient's disturbed consciousness, myoclonus, and parkinsonism were alleviated through rehabilitation therapy.

Brain hypoxia following opiate use has been reported as a rare complication [5-9]. Among them, DHE is extremely rare. As far as we know, there are two reports about DHE caused by the overuse of transdermal fentanyl patches [8,9]. Although the dosage of fentanyl was not specified in these previously reported cases, one case involved the use of more than 10 fentanyl patches, while another described the use of fentanyl patches that were prescribed to someone else for managing cancer-related pain. What is common among these cases, including ours, is that after experiencing consciousness disturbances, the patient undergoes a lucid interval for several days, followed by memory impairment and abnormal behavior. Additionally, white matter lesions in the brain, which were not observed during the initial hospitalization, appeared during the subsequent study. The mechanism behind DHE involves the inhibition of the ATP-dependent enzymatic pathway responsible for producing myelin in cerebral white matter due to hypoxia, which leads to delayed demyelination [10]. However, it remains unclear why DHE occurs following fentanyl overdose. Fentanyl is known to alter oxygen levels in the brain, initially increasing oxygen in brain tissue at low doses, then decreasing oxygen levels at higher doses, and subsequently increasing oxygen levels again at even higher doses [11]. Thus, when exposed to very high doses of fentanyl, brain tissue becomes hyperoxic. We presume that as the concentration decreases to a certain level, the brain tissue may become hypoxic, leading to the development of DHE. Naloxone, a drug used to treat opioid overdose, has been reported as a potential treatment for opioid-induced hypoxic encephalopathy in mice models, and naloxone may be an option for preventing brain hypoxia after fentanyl overdose [12]. However, in our case, naloxone was administered but did not prevent DHE.

Alternately, BPL is an extremely rare complication of opiate. BPL is perhaps most commonly associated with fatal cases of carbon monoxide poisoning [13]. Other reported causes include deaths involving 3,4-methylenedioxymethamphetamine (MDMA), cocaine, opiates, and cyanide poisoning [13,14]. While it has been reported that BPL is found in 5-10% of opiate addicts, there are limited documented reports relating to BPL after substance use [13]. Clinically, globus pallidus lesion typically presents with acute cognitive impairment, coma, or even death, although it can sometimes manifest in a more chronic manner with personality changes or ataxia similar to Parkinson's disease [13,15]. In the literature, there are two possible causes suggested for BPL: cerebral infarction and necrosis. Iqbal et al. reported a case of BPL after heroin inhalation [16]. This case presented with transient disturbance of consciousness and right hemiplegia after inhaling heroin, and BPL was diagnosed with cerebral infarction based on high signal intensity on diffusion-weighted MRI and low ADC values [16]. Zuckerman et al. reported a 17-year-old case of BPL and hypotoxic encephalopathy, which diagnosed cerebral infarction by MRI findings, after halation of heroin [17]. Alternately, Alquist et al. reported an autopsy case of BPL after polysubstance abuse including opiates (oxymorphone), and pathological findings revealed hemorrhage and necrosis in the bilateral globus pallidus [13]. Regarding BPL in our case, based on the MRI findings, most parts of the BPL showed high signal intensity on T2-weighted images, with some marginal areas showing low signal intensity on T2*-weighted images and high signal intensity on T1-weighted images, which were considered consistent with necrosis and hemorrhage. Therefore, we presumed that BPL after opioid overdose is caused by hemorrhagic necrosis rather than ischemic lesions. Various factors can contribute to the onset of BPL, but in our case, carbon monoxide poisoning was ruled out based on the circumstances, and there was no history of other narcotic use. Based on these findings, we concluded that the BPL was caused by fentanyl intoxication.

## Conclusions

We present a case of BPL and DHE following the overuse of transdermal fentanyl patches, confirmed by ruling out other potential causes based on the patient's medical history. The fentanyl had been newly prescribed, and the dosage used was at least 40 mg. Although transdermal fentanyl patches are widely used in clinical settings due to their relatively high efficacy and safety, according to previous reports, fentanyl overdose may lead to DHE and/or BPL. Basic medical studies suggest that naloxone may help improve opioid-induced hypoxic encephalopathy. In our case, the primary cause of BPL was thought to be hemorrhagic necrosis. Further research is needed to elucidate the underlying mechanisms of BPL and/or DHE induced by fentanyl overuse. 
